# Association between quality of life and redo procedures after pulmonary vein isolation in atrial fibrillation patients: Data from the Netherlands Heart Registration

**DOI:** 10.1016/j.hroo.2025.03.017

**Published:** 2025-03-22

**Authors:** Mileen R.D. van de Kar, Gijs J. van Steenbergen, Jasper R. Vermeer, Jeroen F. van der Heijden, Jippe F. Balt, Justin G.L.M. Luermans, Yuri Blaauw, Niki M. Medendorp, Daniela N. Veldman-Schulz, Lukas R.C. Dekker, Dennis van Veghel, Ablation Registration Committee of the Netherlands Heart Registration

**Affiliations:** 1 Heart Centre Catharina Hospital, Eindhoven, the Netherlands; 2 Department of Cardiology, Haga Teaching Hospital, The Hague, the Netherlands; 3 Department of Cardiology, Antonius Hospital, Utrecht, the Netherlands; 4 Department of Cardiology, Cardiovascular Research Institute Maastricht (CARIM), Maastricht University Medical Centre (MUMC+), Maastricht, the Netherlands; 5 Department of Cardiology, University Medical Centre Groningen, Groningen, the Netherlands; 6 Netherlands Heart Registration, Utrecht, the Netherlands; 7 Department of Biomedical Technology, Eindhoven University of Technology, Eindhoven, the Netherlands

**Keywords:** Atrial fibrillation, Pulmonary vein isolation, Quality of life, Real-world data, Patient-centered care, Benchmarking

## Abstract

**Background:**

Atrial fibrillation (AF) significantly impacts quality of life (QoL), necessitating effective therapeutic interventions such as pulmonary vein isolation (PVI). Although PVI’s success is often measured by freedom from arrhythmia, clinical practice emphasizes symptom freedom and patient comfort, as captured by QoL tools such as the Atrial Fibrillation Effect on Quality-of-Life (AFEQT) questionnaire.

**Objective:**

This study investigates the association between baseline QoL, changes in QoL, and redo PVI, aiming to align patient-centered outcomes with clinical decision-making.

**Methods:**

Data from 3336 AF patients undergoing first-time PVI between 2018 and 2021 in Dutch heart centers were analyzed. Patients with baseline and 1-year follow-up QoL scores were categorized into quartiles based on pre-PVI AFEQT scores, and redo PVI rates were assessed within 1 year.

**Results:**

Redo PVI was performed in 16.0% of patients within 1 year. Significant differences in left atrial volume index, left ventricular ejection fraction, and AF type were observed between redo and no-redo groups. Median AFEQT scores improved significantly: from 53.9 to 71.3 in redo patients and from 57.4 to 88.0 in others (*P* < .001). Higher baseline QoL scores correlated with lower redo rates: odds ratios for redo PVI were 0.93 (Q2, *P* =.52), 0.74 (Q3, *P* =.05), and 0.62 (Q4, *P* =.001) compared with Q1. Redo PVI rates varied significantly between heart centers, reflecting practice variation.

**Conclusion:**

Lower baseline QoL is associated with a higher likelihood of redo PVI, emphasizing the role of patient-reported outcomes in AF management. Integrating QoL assessments into routine practice may support individualized care, create risk stratification, and contribute to standardizing clinical decision-making.

## Graphical abstract

**Figure:**
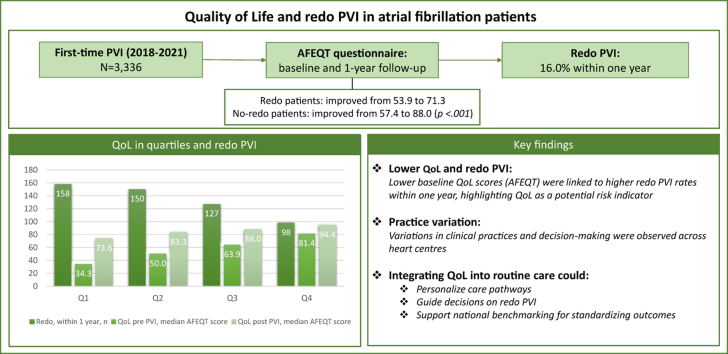

**Key findings**

▪ Quality of Life (QoL) and redo PVI association: Lower baseline QoL scores, as measured by the AFEQT questionnaire, were associated with higher redo PVI rates within 1 year, highlighting the potential role of QoL as a risk indicator for procedural outcomes.
▪ Significant QoL improvements: Median AFEQT scores significantly improved post-PVI, from 53.9 to 71.3 in redo patients and from 57.4 to 88.0 in no-redo patients (*P* < .001), indicating the overall positive impact of PVI on patient-reported outcomes.
▪ Practice variation across heart centers: Redo PVI rates varied significantly between heart centers, reflecting differences in clinical practices and decision-making processes that may influence procedural outcomes.
▪ Clinical implications: Integrating QoL assessments into routine clinical practice could enhance personalized care, guide decision-making for redo PVI, and support national benchmarking efforts to standardize care pathways and improve value-based outcomes.

## Introduction

Atrial fibrillation (AF) is the most prevalent cardiac arrhythmia, significantly impairing the quality of life (QoL) of those affected. Effective therapeutic interventions are necessary to manage the significant burden of AF, including debilitating symptoms such as palpitations, shortness of breath, and fatigue. These interventions aim to control arrhythmia and improve QoL outcomes for patients. Given that symptom relief is a primary goal of AF treatment, QoL assessment has become increasingly recognized as a crucial endpoint in patient management.^1^

One of the primary therapeutic strategies for AF is catheter ablation, particularly pulmonary vein isolation (PVI). The clinical goal of PVI is to create continuous and long-lasting lesions that prevent pulmonary vein reconnection and subsequent arrhythmia recurrence.^1^^,^^2^ Traditionally, successful PVI outcomes are defined by freedom from AF, discontinuation of antiarrhythmic drugs, and avoidance of repeat ablations. However, a more patient-centered approach also considers improvements in QoL as essential markers of success, as highlighted in the European Society of Cardiology 2024 guidelines.^1^ Tools such as the Atrial Fibrillation Effect on Quality-of-Life (AFEQT) questionnaire and symptom diaries are used to assess patient-reported outcomes, giving a more patient-centered evaluation of treatment success.^3^

Although ablation therapy has been shown to improve QoL by reducing AF symptoms, recurrent arrhythmias after PVI often lead to repeat procedures, impacting both patient outcomes and health care utilization.^4^, ^5^, ^6^, ^7^

Unlike initial ablation decisions, which are often guided by objective arrhythmia burden, redo PVI is frequently performed based on symptomatic recurrence, underscoring the importance of QoL assessments in guiding repeat procedures.^1^

Previous studies have demonstrated the positive effects of PVI on QoL and identified factors such as comorbidities that predict the need for repeat ablations.^1^, ^2^, ^3^, ^4^ However, the direct relationship between QoL and redo PVI remains insufficiently understood. Whether lower QoL at baseline, independent of other clinical factors, serves as a predictor for redo procedures remains unclear. Given the high rates of early AF recurrence, which can reduce QoL and prompt consideration of redo PVI, evaluating how QoL changes correlate with redo PVI decisions may provide valuable insights into patient-centered procedural planning.^8^, ^9^, ^10^, ^11^

Addressing this issue is important for optimizing care pathways and ensuring that redo ablation decisions align with patient-centered outcomes and value-based health care principles.^12^ By integrating QoL assessments into routine clinical practice, health care providers could gain a clearer understanding of when redo procedures are truly beneficial for patients. Despite the increasing focus on shared decision-making in AF care (European Society of Cardiology 2024, Class 1C recommendation), current guidelines lack specific guidance on how QoL should be used in redo PVI decision-making.

Therefore, this study aims to assess whether baseline QoL and changes in QoL over time are predictive of undergoing a redo PVI. By doing so, we hope to contribute to advancing AF management by supporting more personalized and well-informed clinical decisions, ultimately fostering better patient outcomes.

## Methods

### Study design and population

This retrospective observational study used data on baseline characteristics and clinical outcomes originating from in-hospital records, which were delivered to and subsequently registered in the Netherlands Heart Registration (NHR). The NHR is a nationwide clinical quality registry and collects patient characteristics, procedure-related data, and outcomes of all invasive cardiac interventions performed in Dutch hospitals. Its overarching aim is to contribute to the preservation and enhancement of the quality and transparency of care for heart patients.^13^ Quality and completeness of data collected by the hospitals are validated by the NHR, amongst others, by means of onsite audits.^14^

The Medical Ethics Board in Nieuwegein, The Netherlands, issued a waiver for informed consent.

All patients who underwent a first PVI as treatment for AF in the Netherlands between 2018 and 2021 (inclusive), and for whom both baseline and 1-year postprocedure QoL questionnaires were available, were included in the study. Patients were included regardless of the ablation technique used.

The study was performed in accordance with the Declaration of Helsinki, and no ethical approval was needed according to the Dutch central committee of Human Research, because the study only used previously collected anonymized cohort registry data. Approval for this study was granted by the Committee of Research and Ethics of the Netherlands Heart Registry on October 30, 2023.

## Baseline patient and procedural characteristics

Baseline and procedural characteristics were selected and defined by the NHR, using a method published earlier.^15^ NHR definitions are in line with the guidelines of the European Society of Cardiology and the American College of Cardiology/American Heart Association.^1^^,^^16^^,^^17^

The following baseline characteristics were collected: age, sex, body mass index (BMI), renal function (measured by estimated glomerular filtration rate [eGFR]), left ventricular ejection fraction (LVEF), left atrial volume index (LAVI), AF type (paroxysmal, persistent, or longstanding persistent), and center of procedure (pseudonymized). These characteristics were chosen based on their reported impact on outcomes after PVI.^5^^,^^18^, ^19^, ^20^

Procedural measures included the PVI method, which encompassed various techniques such as conventional point-by-point radiofrequency ablation, in which radiofrequency energy (RF) is sequentially delivered through a catheter tip to create localized thermal lesions; pulmonary vein ablation catheter RF ablation, using a circular multi-electrode catheter to deliver radiofrequency energy; cryo-balloon ablation, employing a balloon catheter with cryotherapy to freeze and ablate tissue around the pulmonary veins; and pulsed-field ablation, a nonthermal ablative technology delivering electrical pulses through a multielectrode catheter.

### Primary outcomes

The primary outcome was the change (delta) in QoL scores 1 year after the initial intervention, comparing patients who underwent a redo procedure with those who did not.

A redo procedure was defined as any additional ablation procedure performed or scheduled within 1 year (≤365 days) of the initial intervention to treat AF.

QoL was assessed using the AFEQT questionnaire, with 2 key time points: baseline (conducted within 2 months before the initial procedure) and 1-year postprocedure (between 10 and 14 months after the intervention).

Quality of life score entailed the AFEQT score after 1 year and the difference (delta) in score between baseline and after 1 year. The AFEQT is a validated 20-question survey evaluating patients’ perceptions in the 4 domains: AF symptoms, functional impairment, treatment concerns, and treatment satisfaction.^3^ Each question is scored using a 7-point Likert scale, with the overall and subscale scores ranging from 0 (complete AF-related disability) to 100 (no AF-related disability). A change of 5 points or more in the overall AFEQT score was considered clinically significant.^21^

### Secondary outcomes

Secondary outcomes focused on examining baseline characteristics and their relationship with redo rates, along with potential differences in QoL outcomes across heart centers. To assess the influence of these characteristics on redo procedures, the cohort was stratified by heart center, allowing for comparisons in both QoL changes and redo rates at an institutional level.

### Statistical analysis

Descriptive statistics were used to analyze the baseline characteristics of PVI patients for accrual and any discrepancies. The distribution of dependent variables was tested using Q-Q plots and the Kolmogorov-Smirnov test. Bivariate analyses were used to display the correlation between patient characteristics and outcomes and to test for significance.

To explore the primary objective in more detail, patients were divided into quartiles based on their baseline QoL scores. Quartile 1 (Q1) represented those with the lowest preprocedural QoL scores, and Quartile 4 (Q4) represented those with the highest. The quartile boundaries were: Q1 < 43.1, Q2 < 56.5, Q3 < 71.3, and Q4 > 71.3.

Multivariate regression analyses were conducted to assess the influence of patient characteristics, QoL, and procedural center on the occurrence of redo PVI procedures. The multivariate regression model included the following variables: change (Δ) in AFEQT score, AFEQT score in quartiles at baseline (T0_Quartiles), age, BMI, sex, eGFR, LVEF, LAVI, ablation method, and scores from the symptoms subdimension, daily activities subdimension, treatment concern subdimension, and treatment satisfaction subdimension at baseline (T0). These variables were analyzed to assess their relationship with redo rates.

Missing values of baseline characteristics were imputed according to guidelines from the Dutch Journal of Medicine and BMC Medical Research Methodology.^22^^,^^23^ Characteristics missing at random and less than 35% were imputed using 10 iterations and 10 imputations, with both baseline characteristics and outcomes serving as predictors. Outcome variables were not imputed. Metrics assessing imputed data quality (fraction missing info, relative increase variance, and relative efficiency) are provided in the supplemental tables.

## Results

A total of 3336 patients from 9 different heart centers in the Netherlands had QoL data available using the AFEQT questionnaire and were included in the study. Of these, 533 patients (16.0%) underwent a redo PVI within 1 year or were placed on the waiting list for a redo PVI within this timeframe. Most patients underwent cryo-balloon ablation (Table 1).

**Table 1:** Patient characteristics stratified for redo PVI

Patient characteristics	Total	Redo	No-redo	*P*
**Patients,** n **(%)**	3336	533 (16.0)	2803 (84.0)	
**Age,** median (IQR)	64.0 (58.0–70.0)	64.0 (58.0–70.0)	64.0 (58.0–70.0)	.74
**Sex,** n (%)				.80
Male	2144 (64.3)	340 (63.8)	1804 (64.4)	
Female	1192 (35.7)	193 (36.2)	999 (35.6)	
**BMI,** median (IQR)	26.6 (24.5–29.5)	26.8 (24.7–30.0)	26.6 (24.5–29.4)	.16
**eGFR,** median (IQR)	73.6 (63.6–84.4)	73.5 (63.0–83.3)	73.7 (63.8–84.6)	.30
**LVEF,** median (IQR)	55.0 (55.0–55.0)	55.0 (55.0–55.0)	55.0 (55.0–55.0)	<.001
EF >50%, n (%)	2701 (83.2)	411 (80.1)	2290 (83.8)	.12
EF 30%–50%, n (%)	506 (15.6)	95 (18.5)	411 (15.0)	
EF <30%, n (%)	38 (1.2)	7 (1.4)	31 (1.1)	
**LAVI,** median (IQR)	35.0 (29.0–43.0)	37.0 (30.5–46.0)	35.0 (29.0–42.0)	<.001
Normal <29 mL/m^2^, n (%)	571 (23.1)	70 (18.7)	501 (23.9)	.01
Mild 29–33 mL/m^2^, n (%)	495 (20.0)	64 (17.1)	431 (20.6)	
Moderate 34–39 mL/m^2^, n (%)	564 (22.8)	86 (23.0)	478 (22.8)	
Severe >39 mL/m^2^, n (%)	841 (34.0)	154 (41.2)	687 (32.8)	
**CHA_2_DS_2_VASc score,** median (IQR)	2.0 (1.0–3.0)	2.0 (1.0–3.0)	2.0 (1.0–3.0)	.26
**AF-type,** n (%)				<.001
Paroxysmal	2427 (72.8)	342 (64.2)	2085 (74.4)	
Persistent	880 (26.3)	186 (34.9)	694 (24.7)	
Long persistent	29 (.9)	5 (.9)	24 (.9)	
**Methods,** n (%)				.002
Point-by-point RF	986 (29.6)	167 (31.3)	819 (29.2)	
PVAC RF	457 (13.7)	91 (17.1)	366 (13.1)	
Cryo-balloon	1840 (55.2)	262 (49.2)	1578 (56.3)	
Electroporation	38 (1.1)	7 (1.3)	31 (1.1)	
Other	15 (.4)	6 (1.1)	9 (.3)	
**Quality of life (AFEQT score)**				
QoL pre PVI, median (IQR)	56.5 (43.1–71.3)	53.9 (40.7–66.7)	57.4 (43.5–71.3)	<.001
QoL post PVI, median (IQR)	86.1 (69.4–96.3)	71.3 (53.7–88.9)	88.0 (73.2–96.3)	<.001
QoL Δ, median (IQR)	23.2 (8.3–38.0)	13.9 (–.9–30.6)	24.6 (10.2–38.9)	<.001
**QoL changes post PVI**				<.001
QoL decrease, n (%)	261 (7.8)	104 (19.5)	157 (5.6)	
QoL remained the same, n (%)	399 (12.0)	81 (15.2)	318 (11.3)	
QoL increase, n (%)	2676 (80.2)	348 (65.3)	2328 (83.1)	

LVEF categories (n = 3245); LAVI categories (n = 2471).

Δ = difference in QoL; AF = atrial fibrillation; BMI = body mass index; eGFR = estimated glomerular filtration rate; LAVI = left atrial volume index; LVEF = left ventricular ejection fraction; PVI = pulmonal vein isolation; redo within 1 year; QoL = quality of life.

The median LVEF was 55.0 (55.0–55.0) across the entire cohort, as well as in both the redo and no redo groups (*P* < .001). The LAVI of the total cohort was 35.0 mL/m^2^ (interquartile range [IQR], 29.0–43.0), with a significant difference between the redo and no redo groups (37.0 mL/m^2^; IQR: 30.5–46.0 vs 35.0 mL/m^2^; IQR: 29.0–42.0; *P* < .001). The cohort comprised 2427 patients with paroxysmal AF (72.8%), with significantly fewer patients with paroxysmal AF in the redo group compared with the no redo group (342 [64.2%] vs 2085 [74.4%]; *P* < .001). No statistically significant differences were observed in the remaining baseline characteristics.

The primary outcome of this study was the change (delta) in AFEQT QoL score 1 year after PVI, comparing patients who underwent redo PVI with those who did not.

The median pre-PVI AFEQT score for the total cohort was 56.5 (IQR, 43.1–71.3), which increased to 86.1 (IQR, 69.4–96.3) post-PVI. Patients who underwent redo PVI had a significantly lower median pre-PVI QoL score (53.9; IQR, 40.7–66.7) compared with those who did not (57.4; IQR, 43.5–71.3; *P* < .001), with a corresponding post-PVI increase to 71.3 (IQR, 53.7–88.9) and 88.0 (IQR, 73.2–96.3), respectively (*P* < .001).

**Figure 1:**
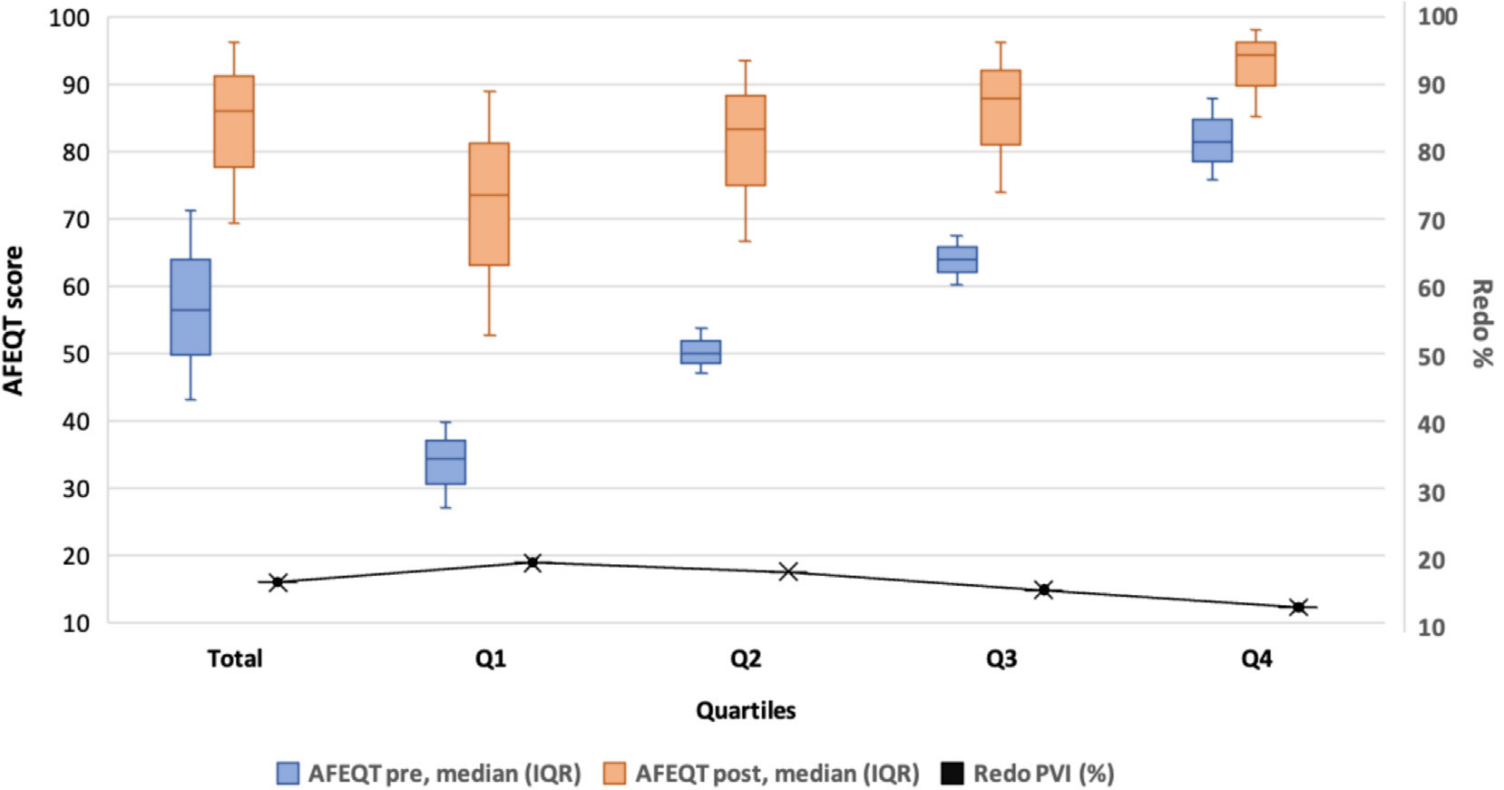
AFEQT scores pre- and post-PVI for quartiles. AFEQT = Atrial Fibrillation Effect on QualiTy-of-life; IQR = interquartile range; PVI = pulmonal vein isolation.

Patients were stratified into quartiles based on the pre-PVI AFEQT scores, with redo rates decreasing progressively across quartiles: Q1 (18.9%), Q2 (17.6%), Q3 (14.9%), and Q4 (12.3%) (*P* < .001) (Figure 1). Baseline characteristics varied significantly across quartiles, with higher QoL quartiles associated with younger age, a higher proportion of males, lower BMI, and higher eGFR (all *P* < .001). For detailed quartile-specific data, please refer to Table 2 and Supplemental Table 1.

**Table 2:** Patient characteristics divided into QoL quartiles at baseline

Patient characteristics	Q1	Q2	Q3	Q4	*P*
**Patients,** n	836	854	852	794	
**Age,** median (IQR)	65.0 (59.0–71.0)	65.0 (59.0–71.0)	64.0 (57.0–70.0)	63.0 (57.0–69.0)	<.001
**Sex,** male, n (%)	442 (52.9)	521 (61.0)	575 (67.5)	606 (76.3)	<.001
**BMI,** median (IQR)	27.4 (24.8–30.6)	26.9 (24.7–30.1)	26.5 (24.4–29.2)	25.9 (24.2–28.3)	<.001
**eGFR,** median (IQR)	72.1 (61.3–83.2)	72.9 (62.5–83.0)	73.7 (64.2–84.9)	75.3 (66.3–86.0)	<.001
**LVEF,** median (IQR)	55.0 (55.0–55.0)	55.0 (55.0–55.0)	55.0 (55.0–55.0)	55.0 (55.0–55.0)	.25
EF >50%, n (%)	658 (80.5)	689 (82.9)	697 (84.0)	657 (85.7)	.09
EF 30%–50%, n (%)	145 (17.7)	132 (15.9)	128 (15.4)	101 (13.2)	
EF <30%, n (%)	14 (1.7)	10 (1.2)	5 (.6)	9 (1.2)	
**LAVI,** median (IQR)	35.0 (29.0–42.0)	35.0 (29.0–42.0)	36.0 (29.0–43.0)	35.0 (29.0–43.0)	.44
Normal <29 mL/m^2^, n (%)	131 (21.3)	151 (24.0)	148 (23.6)	141 (23.5)	.77
Mild 29–33 mL/m^2^, n (%)	130 (21.2)	128 (20.4)	120 (19.1)	117 (19.5)	
Moderate 34–39 mL/m^2^, n (%)	146 (23.8)	151 (24.0)	132 (21.0)	135 (22.5)	
Severe >39 mL/m^2^, n (%)	207 (33.7)	198 (31.5)	228 (36.3)	208 (34.6)	
**AF-type,** n (%)					.26
Paroxysmal	596 (71.3)	630 (73.8)	624 (73.2)	573 (72.2)	
Persistent	229 (27.4)	217 (25.4)	215 (25.2)	216 (27.2)	
Long persistent	9 (1.1)	5 (.6)	12 (1.4)	3 (.4)	

LVEF categories (n = 3245), LAVI categories (n = 2471).

AF = atrial fibrillation; BMI = body mass index; eGFR = estimated glomerular filtration rate; IQR = interquartile range; LAVI, left atrial volume index; LVEF = left ventricular ejection fraction; Q = quartile.

Of the 3336 patients examined, 2676 (80.2%) demonstrated an increase in AFEQT score of ≥5 points, whereas 261 (7.8%) exhibited a decrease of ≥5 points. A strong relationship between pre-PVI QoL quartile and QoL improvement was observed; patients in the lowest quartile (Q1) exhibited a significant improvement in 754 cases (90.2%), whereas 38 patients (4.6%) experienced a decline. In contrast, patients in Q4 (highest baseline QoL) showed a lower likelihood of improvement (62.1%) and a higher frequency of deterioration (13.4%) (*P* < .001).

**Figure 2:**
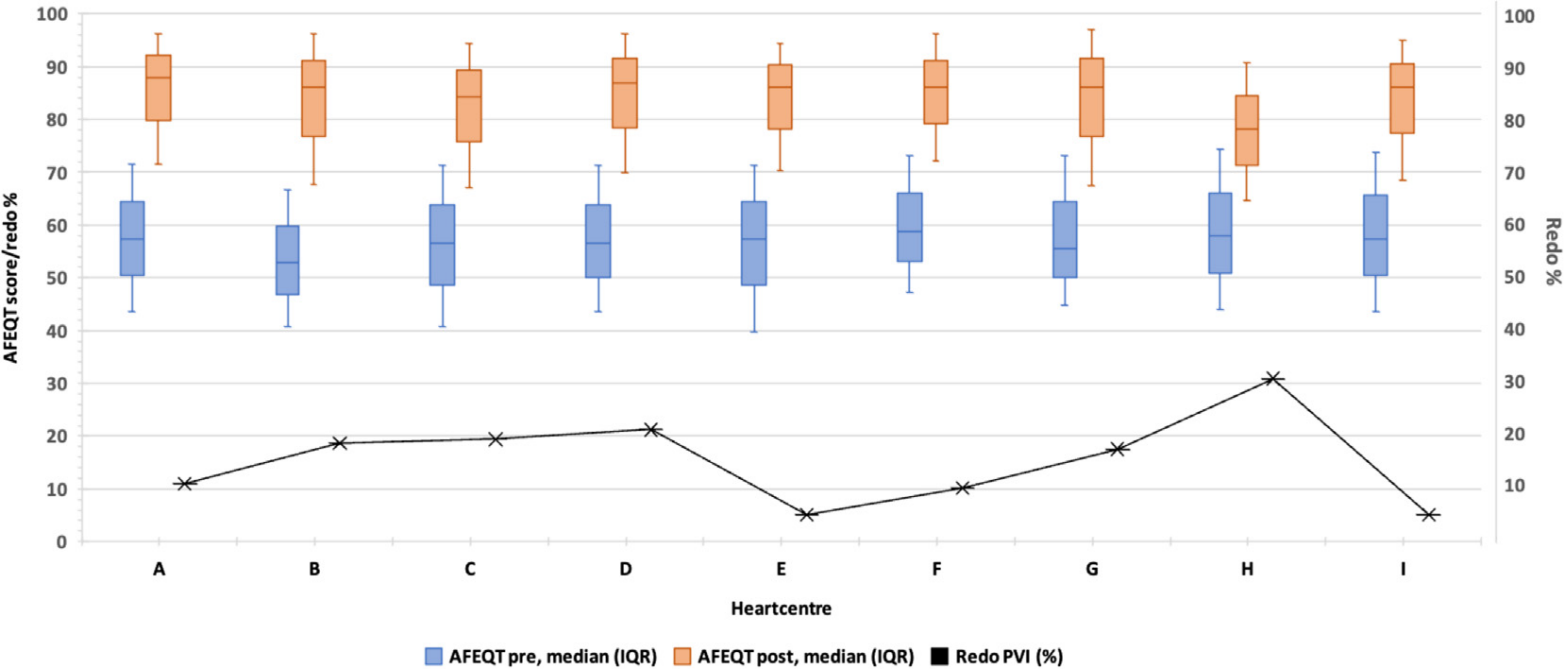
AFEQT scores pre- and post PVI for heart centers. AFEQT = Atrial Fibrillation Effect on QualiTy-of-life; IQR = interquartile range; PVI = pulmonal vein isolation.

A stratified analysis by heart center (Figure 2) revealed significant variation in redo PVI rates across centers (*P* < .001). See also Supplemental Table 2.

A lower baseline QoL score was independently associated with a higher likelihood of redo PVI, with a progressive decrease in redo risk across quartiles (Q2–Q4) compared with Q1 (lowest QoL). The adjusted odds ratios for redo PVI were 0.93 (95% confidence interval [CI], 0.72–1.20; *P* = .57) for Q2, 0.74 (95% CI, 0.57–0.97; *P* = .05) for Q3, and 0.62 (95% CI, 0.47–0.83; *P* = .001), indicating that patients with higher baseline QoL had a significantly lower probability of undergoing redo PVI.

The model was adjusted for age, BMI, sex, eGFR, LVEF, LAVI, AF type, heart center, and ablation method (Table 3; full model details in Supplemental Table 3).

**Table 3:** Multivariate logistic regression analysis of correlation between AFEQT (QoL) and redo PVI

Redo PVI			
Baseline QoL Quartile (T0_AFEQT)	**OR**	**95% CI**	***P***
**Q1** (lowest QoL)	Ref	Ref	Ref
**Q2**	0.93	0.72–1.20	.57
**Q3**	0.74	0.57–0.97	.05
**Q4** (highest QoL)	0.62	0.47–0.83	.001

Odds ratios (OR) for undergoing redo PVI are presented by quartiles of baseline AFEQT scores (T0_AFEQT). Q1 is the reference group (lowest QoL). Higher quartiles (Q2–Q4) correspond to better baseline QoL. Results are adjusted for age, BMI, sex, eGFR, LVEF, LAVI, AF type, heart center, and ablation method.

AF = atrial fibrillation; BMI = body mass index; eGFR = estimated glomerular filtration rate; IQR = interquartile range; LAVI = left atrial volume index; LVEF = left ventricular ejection fraction; Q = quartile.

Beyond QoL, LAVI and AF type were identified as independent predictors of redo PVI, whereas age, BMI, sex, eGFR, and LVEF were not significantly associated with redo risk.

## Discussion

This study offers a comprehensive analysis of real-world data, highlighting the role of QoL in guiding redo PVI decisions across diverse clinical settings. By focusing on patient-centered outcomes, it underscores the utility of integrating QoL assessments into routine AF management.

The study comprised 3336 individuals from 9 Dutch heart centers. Over the course of the year after PVI, 16.0% of patients underwent or were scheduled for a redo PVI procedure. Patients requiring redo procedures showed significant differences in baseline characteristics, particularly in LAVI, AF subtype distribution, and LVEF, compared with those who did not require a redo PVI.

An analysis of pre-PVI AFEQT scores indicated that patients with lower scores were more likely to undergo a redo PVI, suggesting a correlation between QoL and the likelihood of repeat procedures.

The observed correlation between lower pre-PVI AFEQT scores and higher repeat PVI rates may reflect an increased AF burden, including more frequent and prolonged AF episodes, as well as a greater impact on daily functioning and well-being.^24^^,^^25^ The AF burden is a known predictor of outcomes following PVI, because of its association with the extent of left atrial substrate development.^26^ Furthermore, the observed increase in AFEQT scores across quartiles post-PVI highlights the procedure’s favorable impact on QoL, particularly for patients with lower baseline scores.

### Clinical implications of QoL assessment

Although QoL is a key measure of treatment success in AF care, it is not consistently integrated into routine clinical practice. This gap is significant, because the findings suggest that patients with lower QoL scores are at greater risk of requiring a redo PVI. Systematic QoL assessment could provide a more comprehensive evaluation of treatment efficacy from the patient's perspective and guide targeted clinical strategies.

First, early identification and risk stratification of patients with low pre-PVI AFEQT scores could enable clinicians to implement tailored follow-up protocols. For these higher-risk patients, more frequent visits and additional diagnostic evaluations, such as extended rhythm monitoring, may be warranted to detect AF recurrence or progression. Cardiologists performing AF ablation could use these assessments to personalize follow-up schedules, ensuring that patients most at risk of requiring a redo procedure receive timely interventions.^27^ Second, enhanced rhythm monitoring strategies, such as wearable or implantable devices, could allow for earlier detection of recurrent AF episodes and facilitate timely interventions.^28^ Although these objective monitoring strategies provide important insights, QoL assessments uniquely capture the patient’s symptom burden and functional limitations, which may be equally relevant in guiding clinical decisions.

Furthermore, patients with low baseline QoL may benefit from comprehensive support strategies, including the optimization of comorbidities, lifestyle modification programs, or psychological counseling, to enhance overall well-being and potentially reduce the need for redo procedures. By integrating QoL into patient counseling, clinicians may help patients better understand the likelihood of requiring multiple procedures and improve shared decision-making.^29^ Improved communication regarding the likelihood of redo procedures and their associated benefits and risks can help set realistic expectations and strengthen the decision-making process. Finally, incorporating QoL data into routine practice could inform benchmarking efforts by identifying variability in practice patterns and outcomes across centers.^29^ The observed differences in redo rates between centers suggest that procedural decisions may be influenced not only by clinical factors but also by institutional policies and resource allocation. Standardizing QoL assessments across centers could contribute to more consistent decision-making and improved patient management. This would support quality improvement initiatives and contribute to the standardization of care for patients undergoing PVI.

Such strategies advocate for a balanced approach, integrating clinical outcomes with a holistic evaluation of treatment efficacy and patient well-being.

The findings support previous research indicating that PVI improves QoL in AF patients.^30^, ^31^, ^32^ However, the smaller change in AFEQT scores observed among patients undergoing redo procedures compared with prior studies suggests potential differences in the effect of redo PVI on QoL. This could reflect residual structural abnormalities, the psychological impact of repeat procedures, or diminishing returns in QoL improvement because of less severe symptoms being addressed.^27^^,^^33^^,^^34^ Additionally, patient expectations, comorbidities, or the timing of QoL assessments may influence the perceived benefits.^20^^,^^35^ These observations highlight the complexity of AF management and underscore the need for further investigation into factors influencing QoL after redo PVI, including patient selection, adjunctive interventions, and broader determinants of well-being.^27^^,^^33^^,^^34^

Although only 16.0% of patients required a redo PVI, this contrasts with the higher AF recurrence rates reported in literature (30%–35%).^36^^,^^37^ This discrepancy may reflect that many patients avoiding redo procedures experience milder symptoms postablation or transition to less severe or asymptomatic stages of the disease.^32^ Alternatively, practitioners may refrain from recommending a redo because of findings at the initial procedure (eg, excessive fibrosis, technical difficulty, or complications), particularly if QoL improvements after the first procedure were modest.^1^ These observations suggest that the decision to perform a redo is influenced not only by clinical factors but also by perceived benefits for the patient’s QoL.

Further studies are needed to explore whether QoL improvements are primarily driven by reduced AF burden or other factors, such as psychological or placebo effects.^37^^,^^38^ Incorporating QoL assessments into risk stratification models could help clinicians provide more realistic expectations for patients and tailor interventions to prioritize those with the greatest need for improvement. The relationship between QoL and redo PVI rates is already established within 1 year, underscoring the potential for QoL assessments to inform timely interventions.

### Strengths and limitations

This study leverages a comprehensive dataset of real-world data, offering valuable insights into the relationship between QoL and redo PVI. However, certain limitations must be acknowledged. The observational design introduces potential biases, including selection and reporting biases. Additionally, the data were collected from 9 heart centers with available AFEQT data, potentially excluding perspectives from centers without such assessments.

A notable limitation lies in the definition of redo PVI, which includes both completed and planned procedures within a 12-month period. This may introduce variability in data collection timing. Nonetheless, the AFEQT questionnaire reflects patients’ experiences over time, offering valuable insights irrespective of its timing relative to redo PVI. Additionally, the absence of objective data on arrhythmia recurrence and postprocedural management limits the ability to fully assess the relationship between symptom burden, AF recurrence, and the need for redo PVI. Specifically, Holter monitoring, AF burden tracking, post-PVI admissions, and data on antiarrhythmic treatments after PVI were not available in this study. Furthermore, variability in AF monitoring strategies and the lack of standardized definitions across centers present additional challenges in interpreting recurrence patterns.^39^^,^^40^

## Conclusions

This study demonstrates that patients who underwent redo PVI had significantly lower baseline QoL scores and experienced a smaller improvement in QoL post-PVI compared with those who did not require a redo procedure. This highlights the potential role of QoL as a risk indicator for procedural outcomes and the likelihood of repeat intervention.

By integrating systematic QoL assessments into routine practice, clinicians may better identify patients at greater risk for repeat procedures and tailor follow-up strategies accordingly.

These findings provide valuable insights into the relationship between patient-reported outcomes and procedural success, underscoring the importance of a patient-centered approach in AF management. Incorporating QoL into clinical decision-making processes has the potential to refine treatment strategies and improve long-term outcomes for individuals with AF.
