# Supplementary material for: Association between quality of life and redo procedures after pulmonary vein isolation in atrial fibrillation patients: Data from the Netherlands Heart Registration
Source: Heart Rhythm O2. 2025 Mar 22;6(6):745–52. doi: 10.1016/j.hroo.2025.03.017 (PMC12287967; doi:10.1016/j.hroo.2025.03.017)
Supplement: Supplemental Tables [file mmc1.docx]

### Appendix

#### Table A1. Patient outcomes divided into QoL quartiles at baseline.

| **Quality of life (AFEQT score)** | **Q1** | **Q2** | **Q3** | **Q4** | **p-value** |
| --- | --- | --- | --- | --- | --- |
| QoL pre PVI, median (IQR) | 34.3 (27.1-38.9) | 50.0 (47.2-53.7) | 63.9 (60.2-67.6) | 81.4 (75.9-88.0) | <.001 |
| QoL post PVI, median (IQR) | 73.6 (52.8-88.9) | 83.3 (66.6-93.5) | 88.0 (74.0-96.3) | 94.4 (85.2-98.2) | <.001 |
| **QoL changes post PVI** |  |  |  |  | <.001 |
| QoL decrease, n (%) | 38 (4.5) | 44 (5.2) | 73 (8.6) | 106 (13.4) |  |
| QoL remained the same, n (%) | 44 (5.3) | 64 (7.5) | 96 (11.3) | 195 (24.6) |  |
| QoL increase, n (%) | 754 (90.2) | 746 (87.4) | 683 (80.2) | 493 (62.1) |  |
| **Redo**, within 1 year, n (%) | 158 (18.9) | 150 (17.6) | 127 (14.9) | 98 (12.3) | .001 |

** QoL, quality of life; AFEQT, Atrial Fibrillation Effect on QualiTy-of-life.*

#### Table A2. Logistic regression analysis on redo PVI stratified for heart centres.

|  | | **B** | **S.E.** | **Sig.** | **Exp(B)** | **95% C.I. for EXP(B)** | | **Fraction missing info** | **Relative increase variance** | **Relative efficiency** |
| --- | --- | --- | --- | --- | --- | --- | --- | --- | --- | --- |
|  | | | | | | **Lower** | **Upper** |  |  |  |
| **A** | T0_Quartiles (Q2) † | -.28 | .35 | .42 | .76 | .38 | 1.50 | .003 | .003 | 1.00 |
|  | T0_Quartiles (Q3) † | -.27 | .33 | .42 | .76 | .40 | 1.47 | .002 | .002 | 1.00 |
|  | T0_Quartiles (Q4) † | -.42 | .36 | .24 | .66 | .32 | 1.33 | .001 | .001 | 1.00 |
|  | Age | -.01 | .02 | .57 | .99 | .96 | 1.02 | .01 | .01 | 1.00 |
|  | BMI | .03 | .03 | .34 | 1.03 | .97 | 1.09 | .02 | .02 | 1.00 |
|  | Sex | -.16 | .28 | .56 | .85 | .50 | 1.46 | .001 | .001 | 1.00 |
|  | eGFR | -.01 | .01 | .45 | .99 | .98 | 1.01 | .002 | .002 | 1.00 |
|  | LVEF | .02 | .03 | .44 | 1.02 | .97 | 1.07 | .01 | .01 | 1.00 |
|  | LAVI | .02 | .02 | .16 | 1.02 | .99 | 1.05 | .36 | .53 | .97 |
|  | AF-type (persistent) ‡ | .68 | .32 | .04 | 1.96 | 1.04 | 3.71 | .02 | .02 | 1.00 |
|  | AF-type (longstanding-persistent) ‡ | .36 | .84 | .67 | 1.43 | .28 | 7.40 | .01 | .01 | 1.00 |
|  | Ablation method (1) (RF PVAC) ¶ | .17 | 1.13 | .88 | 1.19 | .13 | 10.79 | .01 | .01 | 1.00 |
|  | Ablation method (2) (Cryo) ¶ | -.01 | .27 | .80 | .94 | .56 | 1.58 | .001 | .001 | 1.00 |
|  | Constant | -3.30 | 2.09 | .12 | .04 | .001 | 2.23 | .002 | .002 | 1.00 |
| **B** | T0_Quartiles (Q2) † | -.16 | .27 | .56 | .85 | .50 | 1.45 | .002 | .002 | 1.00 |
|  | T0_Quartiles (Q3) † | -.17 | .28 | .54 | .85 | .49 | 1.46 | .003 | .003 | 1.00 |
|  | T0_Quartiles (Q4) † | -.08 | .31 | .80 | .92 | .50 | 1.70 | .003 | .003 | 1.00 |
|  | Age | -.01 | .01 | .27 | .99 | .96 | 1.01 | .02 | .02 | 1.00 |
|  | BMI | -.03 | .03 | .24 | .97 | .91 | 1.02 | .02 | .02 | 1.00 |
|  | Sex | -.11 | .23 | .64 | .90 | .58 | 1.41 | .003 | .003 | 1.00 |
|  | eGFR | -.003 | .01 | .71 | 1.00 | .98 | 1.01 | .10 | .11 | .99 |
|  | LVEF | -.01 | .02 | .38 | .99 | .96 | 1.02 | .04 | .04 | 1.00 |
|  | LAVI | .02 | .01 | .06 | 1.02 | 1.00 | 1.05 | .13 | .15 | .99 |
|  | AF-type (persistent) ‡ | .28 | .27 | .29 | 1.33 | .79 | 2.24 | .01 | .01 | 1.00 |
|  | AF-type (longstanding-persistent) ‡ | -19.31 | 22728.52 | 1.00 | .00 | .00 | . | .00 | .00 | 1.00 |
|  | Ablation method (1) (RF PVAC) ¶ | .50 | .25 | .05 | 1.64 | 1.00 | 2.70 | .01 | .01 | 1.00 |
|  | Ablation method (2) (Other) ¶ | 23.07 | 17844.80 | 1.00 | 10.4E+8 | .00 | . | .00 | .00 | 1.00 |
|  | Constant | .30 | 1.71 | .86 | 1.35 | .05 | 38.46 | .04 | .04 | 1.00 |
| **C** | T0_Quartiles (Q2) † | 1.05 | .58 | .07 | 2.85 | .91 | 8.89 | .01 | .01 | 1.00 |
|  | T0_Quartiles (Q3) † | .06 | .64 | .92 | 1.07 | .30 | 3.75 | .01 | .01 | 1.00 |
|  | T0_Quartiles (Q4) † | .09 | .65 | .90 | 1.09 | .31 | 3.87 | .01 | .01 | 1.00 |
|  | Age | .01 | .03 | .85 | 1.01 | .95 | 1.07 | .01 | .01 | 1.00 |
|  | BMI | .02 | .05 | .69 | 1.02 | .92 | 1.13 | .01 | .01 | 1.00 |
|  | Sex | -.39 | .48 | .42 | .68 | .27 | 1.73 | .01 | .01 | 1.00 |
|  | eGFR | -.003 | .01 | .80 | 1.00 | .97 | 1.02 | .02 | .02 | 1.00 |
|  | LVEF | .02 | .04 | .56 | 1.03 | .95 | 1.11 | .15 | .17 | .99 |
|  | LAVI | .03 | .02 | .15 | 1.03 | .99 | 1.07 | .17 | .20 | .98 |
|  | AF-type (persistent) ‡ | -.01 | .47 | .99 | .99 | .40 | 2.47 | .01 | .01 | 1.00 |
|  | AF-type (longstanding-persistent) ‡ | -18.52 | 40192.97 | 1.00 | .00 | .00 | . | .00 | .00 | 1.00 |
|  | Ablation method (1) (Cryo) ¶ | 1.10 | .66 | .10 | 2.93 | .84 | 10.73 | .01 | .01 | 1.00 |
|  | Ablation method (2) (Electro) ¶ | 1.30 | .80 | .10 | 3.66 | .93 | 16.89 | .003 | .003 | 1.00 |
|  | Ablation method (3) (Other) ¶ | -.03 | 1.26 | .98 | .98 | .08 | 11.63 | .003 | .003 | 1.00 |
|  | Constant | -5.21 | 3.25 | .11 | .01 | .00 | 3.44 | .03 | .04 | 1.00 |
| **D** | T0_Quartiles (Q2) † | .15 | .27 | .59 | 1.16 | .68 | 1.97 | .003 | .003 | 1.00 |
|  | T0_Quartiles (Q3) † | -.11 | .28 | .70 | .90 | .52 | 1.56 | .004 | .004 | 1.00 |
|  | T0_Quartiles (Q4) † | -.56 | .31 | .07 | .57 | .31 | 1.05 | .002 | .002 | 1.00 |
|  | Age | -.004 | .01 | .73 | 1.00 | .97 | 1.02 | .01 | .01 | 1.00 |
|  | BMI | .04 | .03 | .18 | 1.04 | .98 | 1.09 | .01 | .01 | 1.00 |
|  | Sex | .39 | .22 | .08 | 1.48 | .96 | 2.28 | .003 | .003 | 1.00 |
|  | eGFR | .01 | .01 | .08 | 1.01 | 1.00 | 1.02 | .01 | .01 | 1.00 |
|  | LVEF | -.004 | .02 | .80 | 1.00 | .97 | 1.03 | .05 | .05 | 1.00 |
|  | LAVI | .02 | .01 | .08 | 1.02 | 1.00 | 1.04 | .42 | .68 | .96 |
|  | AF-type (persistent) ‡ | .57 | .21 | .01 | 1.76 | 1.16 | 2.68 | .004 | .004 | 1.00 |
|  | AF-type (longstanding-persistent) ‡ | -.01 | .85 | .99 | .99 | .19 | .24 | .02 | .02 | 1.00 |
|  | Ablation method (1) (Cryo) ¶ | -.46 | 1.12 | .70 | .63 | .06 | 6.48 | .002 | .002 | 1.00 |
|  | Ablation method (2) (Electro) ¶ | -1.78 | 1.58 | .26 | .17 | .01 | 3.74 | .002 | .002 | 1.00 |
|  | Constant | -3.48 | 1.96 | .08 | .05 | .001 | 2.04 | .03 | .03 | 1.00 |
| **E** | T0_Quartiles (Q2) † | -.21.49 | 12954.02 | 1.00 | .00 | .00 | . | .00 | .00 | 1.00 |
|  | T0_Quartiles (Q3) † | -69.13 | 11684.19 | 1.00 | .00 | .00 | . | .00 | .00 | 1.00 |
|  | T0_Quartiles (Q4) † | -23.49 | 14676.65 | 1.00 | .00 | .00 | . | .00 | .00 | 1.00 |
|  | Age | .97 | 467.79 | 1.00 | 2.65 | .00 | . | .00 | .00 | 1.00 |
|  | BMI | 1.03 | 1683.21 | 1.00 | 2.81 | .00 | . | .00 | .00 | 1.00 |
|  | Sex | -60.84 | 10793.77 | 1.00 | .00 | .00 | . | .00 | .00 | 1.00 |
|  | eGFR | 1.43 | 284.43 | 1.00 | 4.20 | .00 | 5.38E+243 | .00 | .00 | 1.00 |
|  | LVEF | 22.96 | 1840.05 | .99 | 9314012581.3 | .00 | . | .00 | .00 | 1.00 |
|  | LAVI | 3.69 | 413.99 | .99 | 39.85 | .00 | . | .00 | .00 | 1.00 |
|  | AF-type (persistent) ‡ | 9.98 | 21514.81 | 1.00 | 21580.16 | .00 | . | .00 | .00 | 1.00 |
|  | Ablation method (1) (Cryo) ¶ | -111.28 | 41533.49 | 1.00 | .00 | .00 | . | .00 | .00 | 1.00 |
|  | Constant | -1646.85 | 147775.18 | .99 | .00 | .00 | . | .00 | .00 | 1.00 |
| **F** | T0_Quartiles (Q2) † | .22 | .56 | .70 | 1.24 | .42 | 3.72 | .004 | .004 | 1.00 |
|  | T0_Quartiles (Q3) † | -.25 | .64 | .70 | .78 | .22 | 2.71 | .01 | .01 | 1.00 |
|  | T0_Quartiles (Q4) † | -.25 | .64 | .70 | .78 | .23 | 2.72 | .003 | .003 | 1.00 |
|  | Age | -.04 | .02 | .11 | .96 | .92 | 1.01 | .004 | .004 | 1.00 |
|  | BMI | .01 | .05 | .91 | 1.01 | .92 | 1.11 | .01 | .01 | 1.00 |
|  | Sex | .60 | .43 | .16 | 1.83 | .79 | 4.24 | .002 | .002 | 1.00 |
|  | eGFR | -.01 | .01 | .69 | 1.00 | .97 | 1.02 | .01 | .01 | 1.00 |
|  | LVEF | -.03 | .04 | .42 | .97 | .89 | 1.05 | .07 | .08 | .99 |
|  | LAVI | -.004 | .03 | .89 | 1.00 | .94 | 1.05 | .49 | .86 | .95 |
|  | AF-type (persistent) ‡ | -.15 | .51 | .77 | .86 | .32 | 2.35 | .02 | .02 | 1.00 |
|  | Ablation method (1) (Cryo) ¶ | -.94 | .89 | .29 | .39 | .07 | 2.23 | .002 | .002 | 1.00 |
|  | Constant | 2.52 | 3.71 | .50 | 12.42 | .01 | 17956.84 | .07 | .07 | .99 |
| **G** | T0_Quartiles (Q2) † | -.40 | .36 | .26 | .67 | .33 | 1.36 | .02 | .02 | 1.00 |
|  | T0_Quartiles (Q3) † | -.99 | .41 | .02 | .37 | .17 | .84 | .02 | .02 | 1.00 |
|  | T0_Quartiles (Q4) † | -2.07 | .52 | <.001 | .13 | .05 | .35 | .02 | .02 | 1.00 |
|  | Age | -.01 | .02 | .70 | .99 | .96 | 1.03 | .03 | .03 | 1.00 |
|  | BMI | -.07 | .04 | .06 | .93 | .86 | 1.00 | .01 | .01 | 1.00 |
|  | Sex | -.09 | .31 | .78 | .92 | .50 | 1.69 | .02 | .02 | 1.00 |
|  | eGFR | -.01 | .01 | .48 | .99 | .98 | 1.01 | .01 | .01 | 1.00 |
|  | LVEF | .04 | .02 | .13 | 1.04 | .99 | 1.08 | .02 | .02 | 1.00 |
|  | LAVI | .04 | .02 | .01 | 1.04 | 1.01 | 1.08 | .28 | .36 | .97 |
|  | AF-type (persistent) ‡ | .37 | .34 | .27 | 1.54 | .74 | 1.33 | .03 | .03 | 1.00 |
|  | AF-type (longstanding-persistent) ‡ | -16.62 | 38130.40 | 1.00 | .00 | .00 | . | .00 | .00 | 1.00 |
|  | Ablation method (1) (RF PVAC) ¶ | 1.14 | .59 | .05 | 3.14 | .99 | 9.98 | .01 | .01 | 1.00 |
|  | Ablation method (2) (Cryo) ¶ | -.31 | .30 | .30 | .73 | .40 | 1.33 | .03 | .03 | 1.00 |
|  | Constant | -1.22 | 2.20 | .58 | .30 | .004 | 22.20 | .03 | .03 | 1.00 |
| **H** | T0_Quartiles (Q2) † | .06 | .52 | .92 | 1.06 | .39 | 2.9 | .00 | .00 | 1.00 |
|  | T0_Quartiles (Q3) † | -.41 | .54 | .45 | .67 | .23 | 1.91 | .002 | .002 | 1.00 |
|  | T0_Quartiles (Q4) † | .13 | .49 | .80 | 1.14 | .44 | 2.96 | .002 | .002 | 1.00 |
|  | Age | .01 | .02 | .60 | 1.01 | .97 | 1.06 | .001 | .001 | 1.00 |
|  | BMI | .02 | .04 | .61 | 1.02 | .94 | 1.11 | .01 | .01 | 1.00 |
|  | Sex | .01 | .40 | .97 | 1.01 | .46 | 2.22 | .001 | .001 | 1.00 |
|  | eGFR | -.02 | .01 | .17 | .98 | .96 | 1.01 | .001 | .001 | 1.00 |
|  | LVEF | -.05 | .03 | .11 | .95 | .90 | 1.01 | .02 | .03 | 1.00 |
|  | LAVI | -.01 | .02 | .84 | 1.00 | .95 | 1.04 | .13 | .15 | .99 |
|  | AF-type (persistent) ‡ | .23 | .41 | .57 | 1.26 | .57 | 2.80 | .01 | .01 | 1.00 |
|  | AF-type (longstanding-persistent)^#^ | 1.23 | 1.67 | .46 | 3.41 | .13 | 90.48 | .03 | .03 | 1.00 |
|  | Ablation method (1)(RF PVAC) ¶ | -21.21 | 40192.97 | 1.00 | .00 | .00 | . | .00 | .00 | 1.00 |
|  | Constant | 1.91 | 3.09 | .54 | 6.74 | .02 | 2856.38 | .01 | .01 | 1.00 |
| **I** | T0_Quartiles (Q2) † | -.34 | .81 | .67 | .71 | .15 | 3.48 | .01 | .01 | 1.00 |
|  | T0_Quartiles (Q3) † | -.56 | .87 | .52 | .57 | .10 | 3.15 | .01 | .01 | 1.00 |
|  | T0_Quartiles (Q4) † | -.55 | .86 | .52 | .58 | .11 | 3.10 | .01 | .01 | 1.00 |
|  | Age | .04 | .04 | .32 | 1.04 | .96 | 1.13 | .01 | .01 | 1.00 |
|  | BMI | .07 | .07 | .37 | 1.07 | .93 | 1.24 | .01 | .01 | 1.00 |
|  | Sex | -.20 | .65 | .75 | .82 | .23 | 2.93 | .01 | .01 | 1.00 |
|  | eGFR | .01 | .02 | .78 | 1.01 | .97 | 1.05 | .07 | .07 | .99 |
|  | LVEF | .06 | .04 | .20 | 1.06 | .97 | 1.15 | .003 | .003 | 1.00 |
|  | LAVI | .04 | .03 | .17 | 1.04 | .99 | 1.09 | .17 | .19 | .98 |
|  | AF-type (persistent) ‡ | 2.16 | .95 | .02 | 8.65 | 1.35 | 55.37 | .003 | .003 | 1.00 |
|  | Ablation method (1) (Cryo) ¶ | 1.42 | .96 | .14 | 4.14 | .64 | 27.04 | .02 | .02 | 1.00 |
|  | Constant | -13.71 | 5.71 | .02 | .00 | .00 | .08 | .06 | .06 | .99 |

*† compared to Q1; ‡ compared to AF-type parxysmal; ¶ compared to ablationmethod RF PBP.*

#### Table A3. Logistic regression analysis on redo PVI with AFEQT at baseline

|  | **B** | **S.E.** | **Sig.** | **Exp(B)** | **95% C.I.for EXP(B)** | | **Fraction missing info** | **Relative increase variance** | **Relative efficiency** |
| --- | --- | --- | --- | --- | --- | --- | --- | --- | --- |
|  |  |  |  |  | **Lower** | **Upper** |  |  |  |
| T0_Quartiles (Q2) † | -0.07 | 0.13 | 0.59 | .93 | .72 | 1.20 | .002 | .002 | 1.00 |
| T0_Quartiles (Q3) † | -.29 | .14 | .03 | .75 | .57 | .98 | .01 | .01 | 1.00 |
| T0_Quartiles (Q4) † | -.49 | .15 | <.001 | .61 | .46 | .82 | .001 | .001 | 1.00 |
| Age | -.01 | .01 | .40 | 1.00 | .98 | 1.01 | .02 | .02 | 1.00 |
| BMI | .002 | .01 | .85 | 1.00 | .98 | 1.03 | .01 | .01 | 1.00 |
| Sex | .03 | .11 | .80 | 1.03 | .83 | 1.27 | .003 | .003 | 1.00 |
| eGFR | -.001 | .003 | .73 | 1.00 | .99 | 1.01 | .04 | .04 | 1.00 |
| LVEF | .001 | .01 | .91 | 1.00 | .99 | 1.02 | .04 | .04 | 1.00 |
| LAVI | .02 | .01 | .002 | 1.02 | 1.01 | 1.03 | .50 | .90 | .95 |
| AF-type (persistent) ^‡^ | .41 | .12 | <.001 | 1.50 | 1.20 | 1.88 | .02 | .02 | 1.00 |
| AF-type (longstanding-persistent) ^‡^ | -.09 | .52 | .86 | .91 | .33 | 2.55 | .04 | .04 | 1.00 |
| Heart centre(B) § | .27 | .23 | .24 | 1.30 | .84 | 2.03 | .002 | .002 | 1.00 |
| Heart centre(C) § | .37 | .25 | .15 | 1.44 | .88 | 2.37 | .002 | .002 | 1.00 |
| Heart centre(D) § | .69 | .17 | <.001 | 2.00 | 1.43 | 2.80 | .004 | .004 | 1.00 |
| Heart centre(E) § | -.73 | .62 | .23 | .48 | .14 | 1.60 | .001 | .001 | 1.00 |
| Heart centre(F) § | -.14 | .24 | .57 | .87 | .54 | 1.40 | .003 | .003 | 1.00 |
| Heart centre(G) § | .49 | .19 | .01 | 1.63 | 1.13 | 2.34 | .002 | .002 | 1.00 |
| Heart centre(H) § | 1.20 | .23 | <.001 | 3.32 | 2.10 | 5.24 | .002 | .002 | 1.00 |
| Heart centre(I) § | -1.01 | .31 | <.001 | .35 | .19 | .64 | .01 | .01 | 1.00 |
| Ablationmethod(1) (RF PVAC) ¶ | .47 | .20 | .02 | 1.60 | 1.07 | 2.38 | .004 | .004 | 1.00 |
| Ablationmethod(2)(Cryo) ¶ | -.05 | .16 | .78 | .96 | .69 | 1.32 | .002 | .002 | 1.00 |
| Ablationmethod(3)(Electro) ¶ | -.02 | .47 | .97 | .98 | .39 | 2.45 | .002 | .002 | 1.00 |
| Ablationmethod(4)(Other) ¶ | 1.28 | .57 | .02 | 3.58 | 1.18 | 10.83 | .001 | .001 | 1.00 |
| Constant | -2.45 | .80 | .002 | .09 | .02 | .41 | .06 | .07 | 1.00 |

*†compared to Q1; ‡ compared to AF-type parxysmal; §compared to Heart centre A; ¶ compared to ablationmethod RF PBP.*

#### Table A4. Members of Ablation Registration Committee of the Netherlands Heart Registration

| **Member** | **Function** | **Centre** |
| --- | --- | --- |
| Mr. W. Kuijt | Cardiologist-Electrophysiologist | Amphia Hospital |
| Dr. A.H.G. Driessen | Cardiothoracic Surgeon | Amsterdam UMC |
| Dr. M. Kemme | Cardiologist-Electrophysiologist | Amsterdam UMC |
| Mr. P.H. van der Voort | Cardiologist-Electrophysiologist | Catharina Hospital |
| Mr. R.E. Bhagwandien | Cardiologist-Electrophysiologist | Erasmus MC |
| Mr. J. van der Heijden | Cardiologist-Electrophysiologist | HagaZiekenhuis |
| Dr. A. Adiyaman | Cardiologist-Electrophysiologist | Isala Hospital |
| Dr. S.A.I.P. Trines | Cardiologist-Electrophysiologist | Leiden University Medical Centre |
| Dr. J.G.L.M. Luermans | Cardiologist-Electrophysiologist | Maastricht UMC+ |
| Dr. B.A. Schoonderwoerd | Cardiologist-Electrophysiologist | Leeuwarden Medical Centre |
| Dr. P.F.H.M. van Dessel | Cardiologist-Electrophysiologist | Medical Spectrum Twente |
| Dr. J.S.S.G. de Jong | Cardiologist-Electrophysiologist | Onze Lieve Vrouwe Gasthuis |
| Mr. S.W. Westra | Cardiologist-Electrophysiologist | Radboudumc |
| Dr. J.C. Balt | Cardiologist-Electrophysiologist | St. Antonius Hospital |
| Dr. R.J. Hassink | Cardiologist-Electrophysiologist | UMC Utrecht |
| Dr Y. Blaauw | Cardiologist-Electrophysiologist | University Medical Centre Groningen |
